# Association between dietary and suicidal behaviors in adolescents in Korea based on the Youth Risk Behavior Survey (2015-2020)

**DOI:** 10.4178/epih.e2022033

**Published:** 2022-03-12

**Authors:** Youngrong Lee, Ye Jin Jeon, Jee-Seon Shim, Sun Jae Jung

**Affiliations:** 1 Department of Preventive Medicine, Yonsei University College of Medicine, Seoul, Korea; 2 Department of Public Health, Yonsei University Graduate School, Seoul, Korea

**Keywords:** Child nutrition sciences, Child psychiatry, Food insecurity, Suicide ideation, Suicide attempt

## Abstract

**OBJECTIVES:**

This study explored the association between dietary and suicidal behaviors of Korean adolescents and investigated differences in this association in children of immigrant parents.

**METHODS:**

The sample (n=368,138) was collected from the Korea Youth Risk Behavior Survey from 2015 to 2020. Participants who agreed to provide family information (n=313,689) were classified according to their parents’ nationality. The study variables were 11 self-reported dietary behaviors, and their composite dietary behaviors (i.e., nutrient deprivation and unhealthy food consumption) that resulted from principal component analysis. The association between study variables and suicide-related outcomes (i.e., suicidal ideation, suicide planning, and suicide attempts) was analyzed by multiple logistic regression with adjustment for covariates. Adjusted odds ratios (aORs) and 95% confidence intervals (CIs) were calculated.

**RESULTS:**

Participants who skipped key meals and foods were more likely to have attemped suicide (aOR [95% CI]: skipping breakfast ≥5 days/wk, 1.28 [1.21 to 1.35]; consuming fruits <1 times/wk: 1.42 [1.32 to 1.52]; consuming vegetables <1 times/wk: 1.72 [1.53 to 1.93]; consuming milk <3 times/wk: 1.07 [0.99 to 1.16]). The associations were prominent in third culture kids (TCKs) (aOR [95% CI]: 2.23 [1.61 to 3.09]; 2.32 [1.61 to 3.35]; 2.63 [1.50 to 4.60]; 1.69 [1.09 to 2.63], respectively). Participants who consumed unhealthy foods (fast food, caffeinated and sugary drinks) more frequently were more likely to have attempted suicide (aOR, 1.55; 95% CI, 1.38 to 1.73). This association was also more prominent in TCKs (aOR, 2.08; 95% CI, 1.08 to 4.01).

**CONCLUSIONS:**

Our findings indicate a positive association between unfavorable dietary behaviors and outcomes related to suicide, and this association appears to be notable in adolescents with immigrant parents.

## GRAPHICAL ABSTRACT

[Fig f3-epih-44-e2022033]

## INTRODUCTION

According to the Global Burden of Disease study, suicide is the leading cause of age-standardized years of life lost in high-income Asia Pacific countries and is in the top 10 leading causes of death in Eastern Europe, Central Asia, southern Latin America, and North America. In individuals between 10 years and 24 years of age, suicide was ranked among the 5 leading causes of mortality in all regions, except for countries in Africa [1]. The early prevalence of suicidal ideation alone or suicidal ideation followed by a suicide attempt is a major risk factor for other psychiatric diagnoses throughout the lifetime, such as major depression, posttraumatic stress disorder, anxiety disorder, attention deficit hyperactivity disorder, and alcohol dependence [2,3]. Survivors of suicide attempts also experience substantial medical and economic burdens that can lead to major health-related costs (e.g., treatment of injuries or permanent disabilities) [4]. Therefore, identifying the causes of suicide in adolescents is a public health priority.

Growing evidence suggests that dietary behaviors (e.g., consumption of fast food, fruit, vegetable, and soft drinks and skipping breakfast) are associated with suicide attempts in adolescents and young adults, notwithstanding commonly known factors such as disadvantaged socioeconomic status [5], parental psychiatric problems, and life-threatening stressors [6]. Recent studies have reported associations between suicide-related outcomes (suicidal ideation, suicide planning, and suicide attempts) and skipping breakfast [7], consumption of fast food [8], and fruit and soft drinks [9]. For example, a large-scale study from 32 countries showed a significant positive association between fast food consumption and suicide attempts (pooled odds ratio [OR], 1.31) in 26 countries [8].

Migration has increased worldwide in recent decades; for instance, the United Nations estimated that the number of international migrants in Europe alone in 2010 was nearly 10% of the European population [10]. Increasingly many Korean men are married to foreign women. In the late 2000s, 8% of marriages in Korea involved wives of foreign origin [11]. Resettling in a new country can be considered a crisis for an individual and their children, which may cause physical and psychological distress. Children of immigrant parents can face challenges in different social roles at home and in society, social marginalization [12], prejudice and discrimination from the host population [13], and economic instability. Studies have also provided evidence that the social and economic disadvantages of immigrants are associated with food insecurity [11], mental illnesses (including depression) [14], and even suicide attempts [15].

To the best of our knowledge, no large-scale studies have examined the relationship between outcomes related to suicide-related outcomes (i.e., suicidal ideation, suicide planning, and suicide attempts) and dietary behavior in Korean adolescents of immigrant parents. Therefore, this study, using Korea Youth Risk Behavior Survey (KYRBS) data obtained through surveys in the 6-year period from 2015 to 2020 (n= 313,689), was conducted to investigate the association between dietary behaviors of Korean adolescents and suicide-related outcomes. An analysis was also conducted to identify differences in this relationship in adolescents with immigrant parents.

## MATERIALS AND METHODS

### Study sample

We recruited 368,138 individuals from the 6-year KYRBS from 2015 to 2020. Among them, 54,449 individuals who did not agree to provide information on their parents’ nationality were excluded, and 313,689 individuals who provided information were selected as the final study sample. The study sample was then classified according to the country of origin of the participants’ parents. If 1 or more of the parents were immigrants, the participants were classified as third-culture kids (TCK), and if both parents were of Korean nationality, they were classified as adolescents with Korean parents. The number of TCKs was 4,925 (1.5%). Participants’ demographic characteristics are summarized in Table 1.

The KYRBS is an anonymous online self-report survey established by the Korea Centers for Disease Control and Prevention (KCDC; now known as the Korea Disease Control and Prevention Agency) and the Ministry of Education. The KYRBS was designed to identify various health behaviors in students aged 12-18 years old. The survey used a multi-stage cluster sampling design to obtain a nationally representative cohort of Korean adolescent students in Korea. In each survey, approximately 60,000 respondents from 800 schools (400 middle schools and 400 high schools) participated. After being informed by trained teachers, students who consented to participate in the study completed the self-administered questionnaire at their school’s computer laboratory. Additional details regarding the sampling methods can be found in the literature [16].

### Dietary behaviors

The predictors comprised 11 dietary behaviors (i.e., consumption of fruits, vegetables, milk, instant noodles, snacks, fast food, water, caffeinated drinks, soft drinks, uncarbonated sugar-rich beverages, and skipping breakfast) based on the criteria of food-based dietary recommendations for Korean adolescents [17]. The KYRBS asked students how often they had engaged in each dietary behavior within the past week, and based on their responses, the participants were divided into 2 groups for all 11 dietary behaviors.

As an example, based on their responses to the item asking about skipping breakfast (excluding milk or juice consumption), participants were categorized as frequently skipping breakfast (skipping 5 or more times a week) or infrequently skipping breakfast (skipping less than 5 times a week). Regarding fast food (e.g., pizza, hamburgers, or fried chicken) and sugar-rich beverage consumption (e.g., carbonated or uncarbonated beverages), participants were divided into frequent (3 or more times a week) and low-frequency consumption groups (fewer than 3 times a week). For fruit (excluding fruit juice) consumption, participants were divided into frequent (once or more per week) and low-frequency consumption groups (less than once per week). Table 2 shows the classification of the dietary behaviors in detail.

As shown in Supplementary Material 1, dietary behaviors, such as skipping breakfast, consumption of soft drinks, fruit, high-sugar drinks (uncarbonated), and fast food were measured in all 6 consecutive years. However, some behaviors were not measured annually. The consumption of caffeinated beverages, vegetables, and dairy was measured over 4 years of the survey (2015-2017 and 2019), while consumption of instant noodles and snacks (2015 and 2017), and water intake (2019 and 2020) were measured in 2 years. Therefore, the analysis excluded samples from the years in which the behavior was not measured (Supplementary Material 1).

Dietary behaviors must be considered as a whole because they are interrelated [18]. The 11 independent variables were items accounting for the behavior of adolescents regarding different kinds of foods. Extracting common behavioral features between these variables and reducing them into a single complex construct that accounts for all of them facilitated an analysis of the association between common dietary behaviors and suicide. To extract features between each dietary variable, principal component analysis (PCA) and confirmatory factor analysis (CFA) were performed to classify each dietary behavior. The results of PCA and CFA are summarized in Supplementary Materials 2 and 3. Based on the results of PCA and CFA, we classified dietary behaviors into nutrient deprivation and unhealthy food consumption. Skipping breakfast and insufficient vegetable, milk, and fruit consumption were classified as nutrient deprivation. Unhealthy food consumption included the variables related to the intake of sugar-rich beverages (including both carbonated and uncarbonated beverages), caffeinated drinks, instant noodles, sweets, and fast food consumption. Water intake was excluded from predictor reduction, because it was not found to be a significant predictor of suicide. The analysis was conducted using the “lavaan” package of R version 3.6.3 (R Core Team, Vienna, Austria).

### Dependent variables

Questions about suicidal ideation, suicide planning, and suicide attempts were assessed with the questions “Have you ever thought about killing yourself?”, “Have you ever planned to kill yourself?” and “Have you ever tried to kill yourself?”, respectively. The options for responses were binary (“yes” or “no”). The number of times participants engaged in suicidal ideation, suicide planning, and suicide attempts was not measured.

### Covariates

As covariates, socio-demographic variables included gender, age (school grade), academic achievement, perceived household income, and type of residence (with family, with relatives, with friends, alone, dormitory, or residence in a facility; dichotomized as with or without family). Perceived academic achievement was divided into 3 groups (high, middle, and low) using a 5-point Likert scale (high, high-middle, middle, low, low-middle, and low). As a proxy variable for socioeconomic status, perceived household income was divided into 3 groups (high, middle, and low) using a 5-point Likert scale (high, high-middle, middle, low, low-middle, and low). Depression has commonly been identified as the most significant clinical risk for suicidal behavior [19,20]. Experience of depressive mood (i.e., “Have you felt sad or desperate to a degree that prevented you from engaging in your usual activities for 2 weeks within the past 12 months?”) was also included as a covariate for analysis.

### Statistical analysis

All statistical analyses were performed using SAS version 9.4 (SAS Institute Inc., Cary, NC, USA). The chi-square test for categorical variables and the independent t-test for continuous variables were used where appropriate to compare demographic features between TCKs and adolescents with Korean parents. Control variables used as covariates were selected based on findings of previous studies. Following the selection of significant covariates, we performed multiple logistic regression analysis to identify relationships between each dietary behavior and suicide-related outcomes (i.e., suicidal ideation, suicide planning, and suicide attempts). Adjusted odds ratios (aORs) and 95% confidence intervals (CIs) were calculated.

### Ethics statement

The Institutional Review Board (IRB) of the KCDC approved the KYRBS (2014-06EXP-02-P-A). Written informed consent was obtained from each participant prior to the survey. Because this web-based survey was performed at schools with a large number of participants, the requirement to obtain informed consent from their parents was exempted by the IRB of the KCDC.

## RESULTS

### Main findings

Table 1 displays the demographic characteristics of the participants (TCKs and adolescents with Korean parents). Approximately a quarter (25.2%) of TCKs skipped breakfast 5 or more days per week, one-third (32.7%) drank soft drinks 3 or more times per week, and approximately 18.0% consumed fast food at least 3 times per week. Adolescents with Korean parents were more likely to consume fast food (19.1%) and drink sugar-sweetened beverages (45.2%) than TCKs, while more TCKs skipped breakfast (25.2%) and consumed more fruit (11.8%) and soft drinks (32.7%). The proportion of respondents drinking caffeinated beverages 3 or more times per week was higher in the TCK group (7.9%), and there was no significant difference in vegetable intake between the 2 groups. The proportion of those consuming milk fewer than 3 times per week was higher in TCKs (5.1%) than in children with Korean parents. The rate of those eating instant noodles (28.9%) or snacks (42.6%) 3 or more times a week was also significantly higher in the TCK group. There was no significant difference in water intake between the 2 groups.

**Figure 1.**
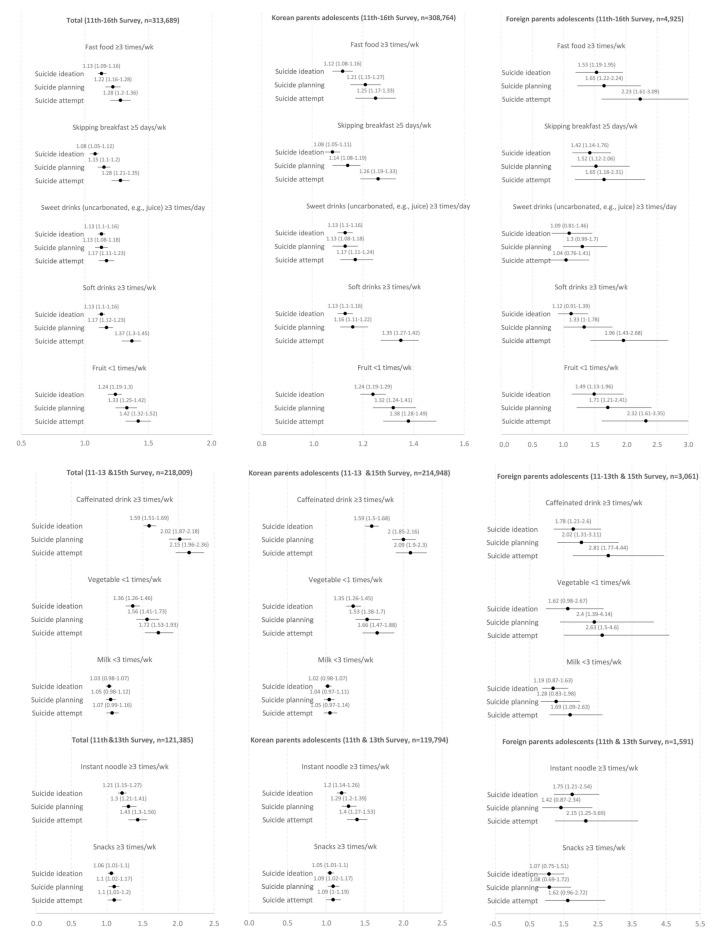
Associations of dietary behaviors with suicidality indices in Korean adolescents in 2015-2020 (n=313,689). Values are presented as adjusted odds ratio (95% confidence interval).

As shown in Table 2 and Figure 1, all dietary behaviors were associated with having experienced depressive mood and suicide-related outcomes (i.e., suicidal ideation, suicide planning, and suicide attempts). The magnitude of the associations tended to increase from suicidal ideation to suicide attempts. Adolescents who consumed fast food 3 or more times per week were more likely to have thought about, planned, and attempted suicide within the last 12 months than adolescents who consumed fast food fewer than 3 times per week (aOR [95% CI]: suicidal ideation, 1.13 [1.09 to 1.16]; suicide planning, 1.22 [1.16 to 1.28]; suicide attempt, 1.28 [1.20 to 1.36]). Adolescents who skipped breakfast were more likely to think, plan and attempt suicide (aOR [95% CI]: 1.08 [1.05 to 1.12]; 1.15 [1.10 to 1.20]; 1.28 [1.21 to 1.35], respectively). A similar tendency was observed in adolescents who consumed fewer than 1 serving of fruit per week (aOR [95% CI]: 1.24 [1.19 to 1.30]; 1.33 [1.25 to 1.42]; 1.42 [1.32 to 1.52], respectively), and consumed soft drinks 3 or more times per week (aOR, [95% CI]: 1.13 [1.10 to 1.16]; 1.17 [1.12 to 1.23]; 1.37 [1.30 to 1.45], respectively). Adolescents who consumed caffeinated beverages 3 or more times per week were more likely to exhibit suicide-related outcomes (aOR [95% CI]: 1.59 [1.51 to 1.69]; 2.02 [1.87 to 2.18]; 2.15 [1.96 to 2.36], respectively). Adolescents who had vegetables fewer than once per week were also more likely to attempt suicide (aOR, 1.72; 95% CI, 1.53 to 1.93), and adolescents who consumed milk fewer than 3 times per week showed similar results (aOR, 1.07; 95% CI, 0.99 to 1.16). Adolescents who consumed instant noodles 3 or more times per week (aOR [95% CI]: 1.21 [1.15 to 1.27]; 1.30 [1.21 to 1.41]; 1.43 [1.30 to 1.56], respectively) and those who had snacks 3 or more times per week (aOR [95% CI]: 1.06 [1.01 to 1.10]; 1.10 [1.02 to 1.17]; 1.10 [1.01 to 1.20], respectively) were more likely to have experienced suicide-related outcomes than their counterparts.

The association between each dietary behavior and the outcomes tended to be greater in TCKs than in adolescents with Korean parents. For all dietary behavior factors except for consumption of sweet drinks, TCKs with unfavorable dietary behavior (i.e., fast food ≥ 3 times/wk, skipping breakfast ≥ 5 days/wk, soft drinks ≥ 3 times/wk, fruits < 1 times/wk) were more likely to think about, plan and attempt suicide than adolescents with Korean parents. For example, TCKs who consumed fast food 3 or more times per week were more likely to have attempted suicide (aOR, 2.23; 95% CI, 1.61 to 3.09) than adolescents with Korean parents (aOR, 1.25; 95% CI, 1.17 to 1.33).

**Figure 2.**
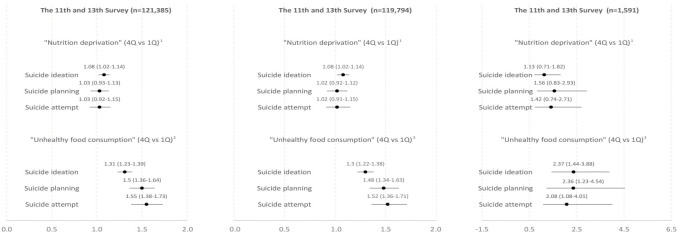
Associations of composite dietary behaviors indices with suicidality in Korean adolescents. Values are presented as adjusted odds ratio (95% confidence interval). ^1^“Nutrient deprivation” score [range, 4 to 29] is composite score comprised of breakfast skipping [1 to 8], and vegitable, milk, and fruit deprivation [1 to 7] as a result of principal component analysis analysis. ^2^“Unhealthy food consumption” score [range, 6 to 36] is composite score comprised of carbonated [1 to 7] and uncarbonated sugar-based beverage [1 to 7], and consumption of instant noodle, caffeinated drink, snacks and fast food [1 to 7], as a result of PCA analysis. Estimates are the odds ratio of upper 25% group and lower 25% group.

As shown in Figure 2 and Supplementary Material 4, composite dietary behavior scores (i.e., nutrient deprivation and unhealthy food consumption) were associated with higher odds of suiciderelated outcomes. The magnitude of the association was obtained by calculating the OR of the top and bottom quarters of composite dietary behavior scores (i.e., nutrient deprivation and unhealthy food consumption). The first composite dietary behavior score (nutrient deprivation), was a composite index of breakfast skipping and intake of fruit, vegetable, and milk. Adolescents with a high frequency of skipping key meals were more likely to experience suicidal ideation; however, the associations were not significant for suicide planning and attempts (aOR [95% CI]: 1.08 [1.02 to 1.14], 1.03 [0.93 to 1.13], 1.03 [0.91 to 1.15], respectively). The other composite dietary behavior score (unhealthy food consumption) was a composite index of consumption of fast food, sugar-rich beverages, caffeinated drink, instant noodles, snacks, and carbonated beverages. A higher number of servings of unhealthy foods consumed per week was associated with higher rates of suicide-related outcomes (aOR [95% CI]: 1.31 [1.23 to 1.39], 1.50 [1.36 to 1.64], 1.55 [1.38 to 1.73], respectively). Similarly, this association tended to be more prominent in TCKs than in adolescents with Korean parents.

## DISCUSSION

In the cohort of more than 300,000 individuals 12-18 years old, we found that suicidal ideation, suicide planning, and suicide attempts were more prevalent in adolescents with unfavorable dietary behaviors (i.e., consuming more sugar-based beverages, fewer fruits, and skipping key meals). Most importantly, we also found that these associations were more prominent in TCKs. Our findings are in line with those of previous cross-sectional studies that investigated the relationship between dietary behaviors (i.e., consumption of sugar-based beverages, fruit, and fast food and skipping breakfast) and outcomes related to suicide. For example, a multinational study including more than 100,000 adolescents from 32 countries found that those who frequently consumed sugary soda were more likely to have attempted suicide than those who less frequently drank soda [9]. In addition, a study of the same population reported that students who consumed fast food more frequently (pooled OR, 1.31; 95% CI, 1.17 to 1.46) were more likely to have attempted suicide [8] than students who consumed fast food less frequently. Furthermore, a study on 62,276 Korean adolescents 12-18 years old reported that frequent (> 5 times/wk) breakfast-skipping adolescents were more prone to attempt suicide than those who infrequently skipped breakfast (≤ 5 times/wk) [7].

Several hypotheses may explain the association between dietary behaviors and suicide-related outcomes. The association between these 2 behaviors can be explained by systemic inflammation in response to a poor diet (i.e., high glycemic index, saturated fats, trans-fatty acids) [21,22], which has recently been discovered to play a role as a predictor of suicide attempts [21,23]. As evidence for this possibility, first, a meta-analysis of studies on children and adolescents aged 2-19 years [24] found that a good-quality diet (high intake of vegetables and fruits, or whole grains) that is rich in macro-/micro-nutrients such as dietary fiber, vitamin C and vitamin E, and unsaturated fats ameliorated low-grade inflammation. Second, a cohort study of 419,527 Korean men and women showed that systemic inflammation was positively associated with the risk of suicide [25]. A recent meta-analysis of 18 studies with 1,743 patients and healthy controls reported that levels of inflammatory markers (i.e., interleukin [IL]-1β and IL-6) in the blood, cerebrospinal fluid, and brain were significantly different between suicidal and non-suicidal individuals [26]. Since variables such as social support, standard of living, and comorbidities may influence suicide [26,27], it is possible that dietary behaviors account for the suicide-related outcomes of this study, mediated by systemic inflammation in response to a poor diet. This is an area of research that needs to be explored in the future.

Alternatively, it is also possible that the relatively prominent associations found between dietary behaviors and suicide attempts in TCKs can be explained by underlying food insecurity. A systemic review of 23 relevant articles reported that members of immigrant households experience considerable psychological distress due to adaptation stress [12] and are at risk of food insecurity [28]. A systematic review of 29 studies reported that food insecurity and acculturation were important social factors influencing dietary habits and contributing to the development of morbidities, which can cause progressive unsustainability of health systems. [29] Another review also concluded that greater consumption of fruits, vegetables, and dairy products, and lower consumption of sugary sweetened beverages and energy-dense foods were associated with better parental socioeconomic status, education, and migrant status [30].

The major strengths of this study include its large representative sample size of Korean adolescents and adolescents with immigrant parents. To the best of our knowledge, this is one of the few large-scale studies analyzing the association between dietary behaviors and outcomes related to suicide in Korean adolescents and those with immigrant parents. This study provides additional data regarding the mental health issues (i.e., suicide) faced by adolescents who might have a constant fear of social discrimination and are underprivileged. In addition, by using the methodology of dimensionality reduction (PCA), we were able to consolidate multiple dietary behaviors into 2 complex constructs that accounted for common features, and to confirm the association between the common features of variables and suicidal behaviors. We also presented rare data that provide insights on the mental health of adolescents with immigrant parents, a population that is increasing in Korea.

However, the present findings should be interpreted with respect to several limitations. First, the study variables were items of a self-reported questionnaire, which may have been subject to measurement errors. There may have been bias in the responses of adolescents who had experienced suicidal behaviors to items related to dietary behaviors, and thus the study estimates may have been overestimated. Second, the non-overlapping timeframe between outcomes related to suicide (past 12 months) and dietary behaviors (past 7 days) should be interpreted with caution. Third, the adolescents included in our study were attending school at the time of the survey; therefore, our findings may not be extrapolated to the general population. Parents’ nationality was analyzed using a self-reported questionnaire item. There may have been measurement errors, potentially leading to an underestimated proportion of TCK. A significant number of adolescents did not consent to the provision of family information and were excluded from the analysis. The true number of immigrant children may thus have been higher. Finally, as the cross-sectional design of the study cannot inform us on the causality of the associations, bidirectional or even reverse associations cannot be ruled out.

Despite possible associations with other unmeasured variables, our data indicate that adolescents with a higher level of consumption of unhealthy foods (i.e., sugar-sweetened and caffeinated drinks and energy-dense fast foods) were more likely to have experienced suicide-related outcomes. We also found that this association was more prominent in children of immigrant parents. Further longitudinal studies should investigate the directionality of this relationship. Public educational institutions and health authorities should employ interventional efforts to improve dietary behaviors and to prevent mental health issues, such as depression and suicide. Possible interventions would include efforts to reduce the consumption of high-sugar drinks and fast food, to decrease the frequency of breakfast skipping, and to increase the consumption of fruits rich in fiber and vitamins.

## Figures and Tables

**Figure f3-epih-44-e2022033:**
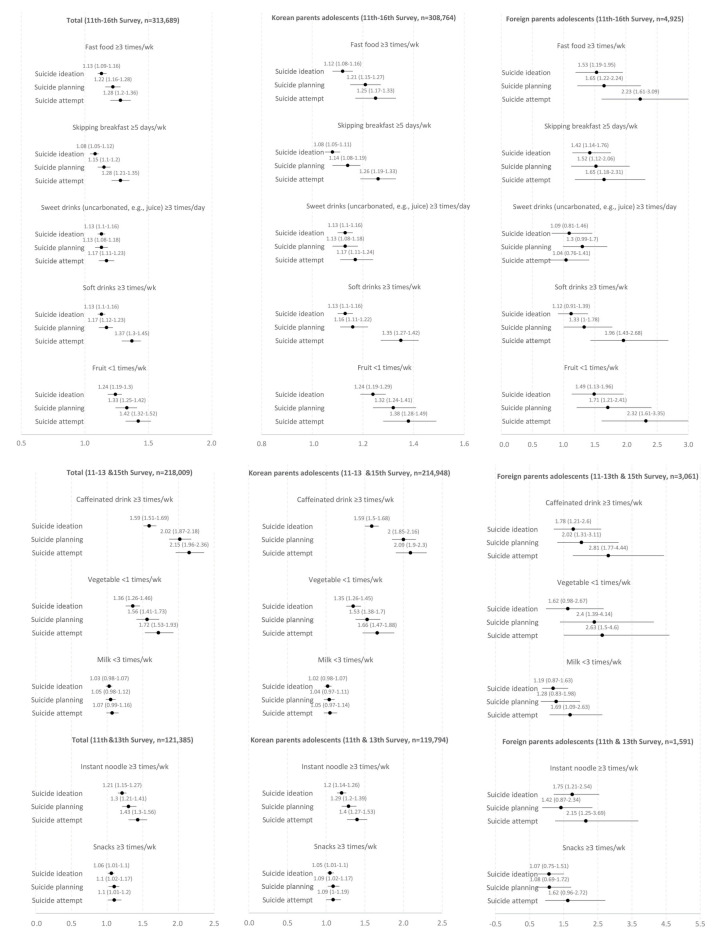


**Table 1.** Demographic and health characteristics of Korean adolescents in 2015-2020^1^

Characteristics			Total	Korean parents	Immigrant parents	p-value
Total, n			313,689	308,764	4,925	
Gender						0.027
	Men		157,635 (50.2)	155,237 (50.3)	2,398 (48.7)	
	Women		156,054 (49.7)	153,527 (49.7)	2,527 (51.3)	
School grade						<0.001
	Middle school		161,866 (51.6)	158,713 (51.4)	3,153 (64.0)	
	High school		151,823 (48.4)	150,051 (48.6)	1,772 (36.0)	
Age, mean ± SD (yr)			14.95±1.75	14.96±1.75	14.52±1.74	
Socioeconomic status^2,3^						<0.001
	High		124,838 (39.8)	123,601 (40.0)	1,237 (25.1)	
	Middle		148,137 (47.2)	145,690 (47.2)	2,447 (49.7)	
	Low		40,714 (13.0)	39,473 (12.6)	1,241 (0.4)	
Family cohabitation^2^						<0.001
	With family		300,096 (95.7)	295,555 (95.7)	4,541 (92.2)	
	Apart from family		13,593 (4.3)	13,209 (4.3)	384 (7.8)	
Academic achievement^2,3^						<0.001
	High		123,405 (39.3)	121,898 (39.5)	1,507 (30.6)	
	Middle		91,381 (29.1)	89,977 (29.1)	1,404 (28.5)	
	Low		98,903 (31.5)	96,889 (30.9)	2,014 (0.6)	
Dietary behaviors^4^						
	KYRBS 2015-2020, total		313,689	308,764 (98.4)	4,925 (1.6)	
		Skipping breakfast (≥5 days/wk)	-	71,508 (23.2)	1,243 (25.2)	0.001
		Fruits (<1 times/wk)	-	29,332 (9.5)	581 (11.8)	<0.001
		Fast food (≥3 times/wk)	-	59,035 (19.1)	887 (18.0)	0.049
		Soft drinks (≥3 times/wk)	-	96,607 (31.3)	1,611 (32.7)	0.033
		Sweet drinks, uncarbonated (≥3 times/wk)	-	139,524 (45.2)	2,082 (42.3)	<0.001
	KYRBS 2015-2017, 2019, total		218,009	214,948 (98.6)	3,061 (1.4)	
		Caffeinated drinks (≥3 times/wk)	-	12,179 (5.7)	241 (7.9)	<0.001
		Vegetables (<1 times/wk)	-	31,450 (14.6)	437 (14.3)	0.581
		Milk (<3 times/wk)	-	9,296 (4.3)	156 (5.1)	0.037
	KYRBS 2015, 2017, total		121,385	119,794 (98.7)	1,591 (1.3)	
		Instant noodles (≥3 times/wk)	-	28,583 (23.9)	460 (28.9)	0.001
		Snacks (≥3 times/wk)	-	46,376 (38.7)	678 (42.6)	<0.001
	KYRBS 2019-2020, total		75,442	73,746 (97.7)	1,696 (2.2)	
		Water (<3 times/day)	-	57,350 (77.8)	1,310 (77.2)	0.606

Values are presented as number (%). SD, standard deviation; KYRBS, Korea Youth Risk Behavior Survey.

1 The sample merged survey data from 6 years between 2015 and 2020, each of which included about 60,000 participants.

2 The variable was obtained by a self-report questionnaire.

3 The variable was coded with 5 options from “high” to “low.” “High” and “moderately high” were classified as “high,” “middle” as “middle,” and “moderately low” and “low” as “low.”

4 Each dietary behavior was set according to the criteria of the food-based dietary recommendations for Korean adolescents [16].

**Table 2.** Associations of each dietary behavior with suicidal-related outcomes^1^ in Korean adolescents in 2015-2020

Variables		Fast food (≥3 times/wk)	Skipping breakfast (≥5 days/wk)^2^	Sweet drinks (≥3 times/day)	Soft drinks (≥3 times/wk)	Fruits (<1 times/wk)	Caffeinated drinks (≥3 times/wk)	Vegetables (<1 times/wk)	Milk (<3 times/wk)	Instant noodles (≥3 times/wk)	Snacks (≥3 times/wk)	Water (<3 times/day)
Total, n		313,689					218,009			121,385		75,442
	Depression, past 12 mo	1.45 (1.42, 1.48)	1.22 (1.20, 1.25)	1.34 (1.32, 1.37)	1.33 (1.31, 1.36)	1.16 (1.13, 1.20)	1.20 (1.14, 1.27)	1.01 (0.98, 1.04)	2.04 (1.96, 2.13)	1.19 (1.15, 1.22)	1.35 (1.31, 1.40)	1.09 (1.05, 1.14)
	Suicidal ideation, past 12 mo	1.13 (1.09, 1.16)	1.08 (1.05, 1.12)	1.13 (1.10, 1.16)	1.13 (1.10, 1.16)	1.24 (1.19, 1.30)	1.59 (1.51, 1.69)	1.36 (1.26, 1.46)	1.03 (0.98, 1.07)	1.21 (1.15, 1.27)	1.06 (1.01, 1.10)	1.03 (0.97, 1.09)
	Suicide planning, past 12 mo	1.22 (1.16, 1.28)	1.15 (1.10, 1.20)	1.13 (1.08, 1.18)	1.17 (1.12, 1.23)	1.33 (1.25, 1.42)	2.02 (1.87, 2.18)	1.56 (1.41, 1.73)	1.05 (0.98, 1.12)	1.30 (1.21, 1.41)	1.10 (1.02, 1.17)	0.98 (0.89, 1.09)
	Suicide attempt, past 12 mo	1.28 (1.20, 1.36)	1.28 (1.21, 1.35)	1.17 (1.11, 1.23)	1.37 (1.30, 1.45)	1.42 (1.32, 1.52)	2.15 (1.96, 2.36)	1.72 (1.53, 1.93)	1.07 (0.99, 1.16)	1.43 (1.30, 1.56)	1.10 (1.01, 1.20)	0.90 (0.79, 1.02)
Adolescents with Korean parents, n		308,764					214,948			119,794		73,746
	Depression, past 12 mo	1.45 (1.42, 1.48)	1.22 (1.19, 1.24)	1.34 (1.32, 1.37)	1.33 (1.30, 1.36)	1.15 (1.12, 1.19)	1.20 (1.14, 1.27)	1.01 (0.98, 1.04)	2.03 (1.94, 2.12)	1.19 (1.15, 1.22)	1.35 (1.30, 1.40)	1.09 (1.04, 1.13)
	Suicidal ideation, past 12 mo	1.12 (1.08, 1.16)	1.08 (1.05, 1.11)	1.13 (1.10, 1.16)	1.13 (1.10, 1.16)	1.24 (1.19, 1.29)	1.59 (1.50, 1.68)	1.35 (1.26, 1.45)	1.02 (0.98, 1.07)	1.20 (1.14, 1.26)	1.05 (1.01, 1.10)	1.03 (0.97, 1.10)
	Suicide planning, past 12 mo	1.21 (1.15, 1.27)	1.14 (1.08, 1.19)	1.13 (1.08, 1.18)	1.16 (1.11, 1.22)	1.32 (1.24, 1.41)	2.00 (1.85, 2.16)	1.53 (1.38, 1.70)	1.04 (0.97, 1.11)	1.29 (1.20, 1.39)	1.09 (1.02, 1.17)	0.98 (0.89, 1.09)
	Suicide attempt, past 12 mo	1.25 (1.17, 1.33)	1.26 (1.19, 1.33)	1.17 (1.11, 1.24)	1.35 (1.27, 1.42)	1.38 (1.28, 1.49)	2.09 (1.90, 2.30)	1.66 (1.47, 1.88)	1.05 (0.97, 1.14)	1.40 (1.27, 1.53)	1.09 (1.00, 1.19)	0.89 (0.79, 1.02)
Adolescents with immigrant parents, n		4,925					3,061			1,591		1,696
	Depression, past 12 mo	1.51 (1.26, 1.80)	1.50 (1.27, 1.76)	1.34 (1.16, 1.55)	1.44 (1.24, 1.68)	1.55 (1.25, 1.94)	1.25 (0.83, 1.90)	1.15 (0.90, 1.47)	2.98 (2.21, 4.00)	1.11 (0.86, 1.43)	1.86 (1.42, 2.44)	1.53 (1.14, 2.06)
	Suicidal ideation, past 12 mo	1.53 (1.19, 1.95)	1.42 (1.14, 1.76)	1.09 (0.81, 1.46)	1.12 (0.91, 1.39)	1.49 (1.13, 1.96)	1.78 (1.21, 2.60)	1.62 (0.98, 2.67)	1.19 (0.87, 1.63)	1.75 (1.21, 2.54)	1.07 (0.75, 1.51)	0.75 (0.49, 1.15)
	Suicide planning, past 12 mo	1.65 (1.22, 2.24)	1.52 (1.12, 2.06)	1.30 (0.99, 1.70)	1.33 (1.00, 1.78)	1.71 (1.21, 2.41)	2.02 (1.31, 3.11)	2.40 (1.39, 4.14)	1.28 (0.83, 1.98)	1.42 (0.87, 2.34)	1.08 (0.69, 1.72)	0.82 (0.46, 1.46)
	Suicide attempt, past 12 mo	2.23 (1.61, 3.09)	1.65 (1.18, 2.31)	1.04 (0.76, 1.41)	1.96 (1.43, 2.68)	2.32 (1.61, 3.35)	2.81 (1.77, 4.44)	2.63 (1.50, 4.60)	1.69 (1.09, 2.63)	2.15 (1.25, 3.69)	1.62 (0.96, 2.72)	1.04 (0.55, 1.94)

Values are presented as adjusted odds ratio (95% confidence interval).

1 Depression was adjusted for sex, school grade, socioeconomic status, academic achievement, and residential status; 3 Models were adjusted for sex, school grade, socioeconomic status, academic achievement, residential status, and experience of depressed mood in the past 12 months.

2 Uncarbonated, e.g., juice.

## SUPPLEMENTARY MATERIALS

Supplementary Material 1. Composition of dietary behavior predictors measured each year

Supplementary Material 2 Result of principle component analysis, Result of confirmatory factor analysis

Supplementary Material 3

Supplementary Material 4 Associations of composite variable of dietary behaviours and suicidal behavriours in Korean adolescents in 2015, and 2017 (N=121,385)
