# Supplementary figures and images for: Association between dietary and suicidal behaviors in adolescents in Korea based on the Youth Risk Behavior Survey (2015-2020)
Source: Epidemiol Health. 2022 Mar 12;44:e2022033. doi: 10.4178/epih.e2022033 (PMC9350419; doi:10.4178/epih.e2022033)

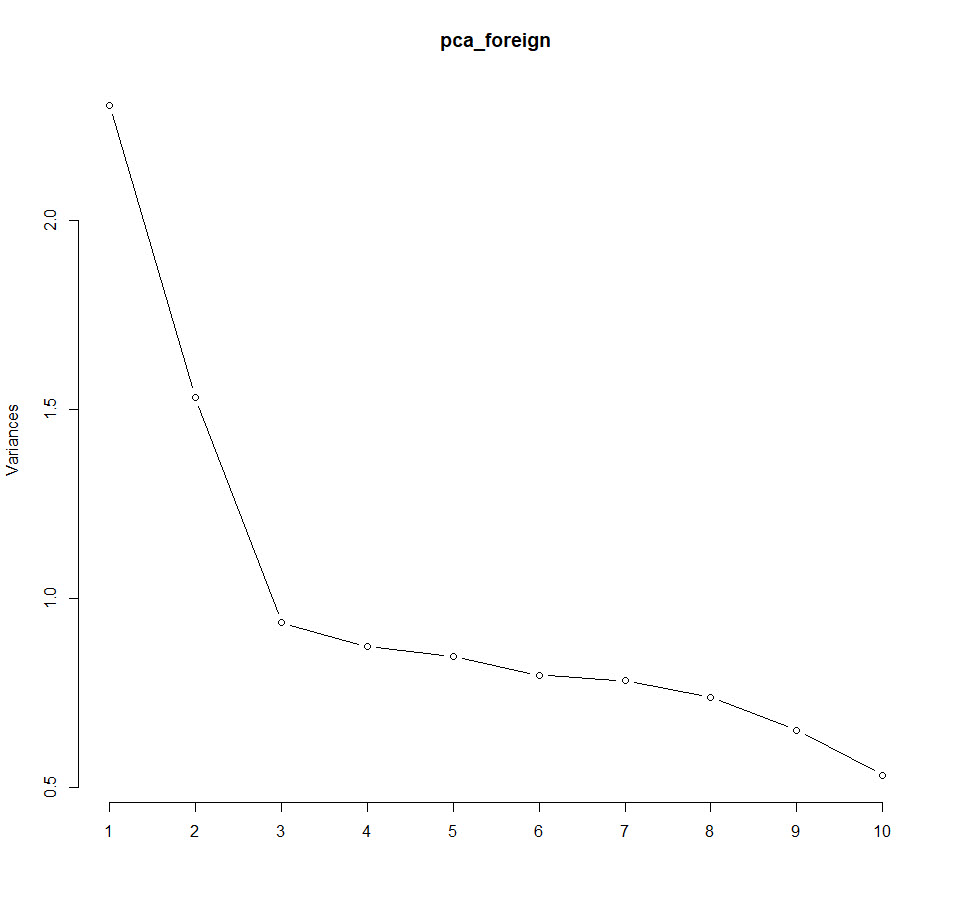

Supplement: Supplementary Material 3 [file epih-44-e2022033-suppl3.tif]
