# Supplementary material for: Association between dietary and suicidal behaviors in adolescents in Korea based on the Youth Risk Behavior Survey (2015-2020)
Source: Epidemiol Health. 2022 Mar 12;44:e2022033. doi: 10.4178/epih.e2022033 (PMC9350419; doi:10.4178/epih.e2022033)
Supplement: Supplementary Material 4 — Associations of composite variable of dietary behaviours and suicidal behavriours in Korean adolescents in 2015, and 2017 (N=121,385) [file epih-44-e2022033-suppl4.docx]

| <Supplementary Material 4. Associations of composite variable of dietary behaviours and suicidal behavriours in Korean adolescents in 2015, and 2017 (N=121,385)> | | | | | | |
| --- | --- | --- | --- | --- | --- | --- |
|  | Depression past 12 months | | | Suicide ideation past 12 months | | |
|  | Total (n=121,385) | Korean parents adolescents (n=119,794) | Foreign parents adolescents (n=1,591) | Total (n=121,385) | Korean parents adolescents (n=119,794) | Foreign parents adolescents (n=1,591) |
|  | AOR (95% CI) | AOR (95% CI) | AOR (95% CI) | AOR (95% CI) | AOR (95% CI) | AOR (95% CI) |
| "Nutrition deprivation" (2Q vs 1Q) ^a^ | 1.10 (1.06-1.14) | 1.10 (1.06-1.14) | 1.27 (0.90-1.79) | 1.07 (1.01-1.13) | 1.06 (1.01-1.12) | 1.56 (1.00-2.46) |
| "Nutrition deprivation" (3Q vs 1Q) ^a^ | 1.17 (1.12-1.23) | 1.17 (1.12-1.23) | 1.33 (0.92-1.93) | 1.18 (1.11-1.25) | 1.18 (1.11-1.25) | 1.65 (1.00-2.71) |
| "Nutrition deprivation" (4Q vs 1Q) ^a^ | **1.33 (1.28-1.39)** | **1.33 (1.27-1.38)** | **1.80 (1.28-2.53)** | **1.30 (1.23-1.38)** | **1.29 (1.22-1.37)** | **1.97 (1.26-3.06)** |
| "Unhealthy food consumption" (2Q vs 1Q) ^b^ | 1.16 (1.11-1.21) | 1.16 (1.11-1.21) | 1.22 (0.87-1.73) | 1.09 (1.03-1.15) | 1.09 (1.03-1.15) | 1.27 (0.79-2.06) |
| "Unhealthy food consumption" (3Q vs 1Q) ^b^ | 1.36 (1.30-1.42) | 1.36 (1.30-1.42) | 1.33 (0.92-1.93) | 1.28 (1.20-1.35) | 1.28 (1.20-1.36) | 1.20 (0.72-1.97) |
| "Unhealthy food consumption" (4Q vs 1Q) ^b^ | **1.90 (1.81-1.98)** | **1.89 (1.81-1.98)** | **2.10 (1.47-2.98)** | **1.76 (1.66-1.86)** | **1.74 (1.65-1.85)** | **2.88 (1.85-4.48)** |
| "Nutrition deprivation" + "Unhealthy food consumption"  (2Q vs 1Q) | 1.14 (1.09-1.19) | 1.14 (1.09-1.19) | 1.43 (0.99-2.07) | 1.13 (1.07-1.20) | 1.12 (1.06-1.19) | 1.89 (1.15-3.11) |
| "Nutrition deprivation" + "Unhealthy food consumption"  (3Q vs 1Q) | 1.36 (1.30-1.41) | 1.35 (1.30-1.41) | 1.86 (1.30-2.65) | 1.28 (1.22-1.35) | 1.27 (1.21-1.34) | 2.19 (1.35-3.54) |
| "Nutrition deprivation" + "Unhealthy food consumption"  (4Q vs 1Q) | **1.88 (1.80-1.97)** | **1.88 (1.79-1.96)** | **2.50 (1.73-3.59)** | **1.84 (1.74-1.94)** | **1.82 (1.73-1.93)** | **3.39 (2.09-5.49)** |
|  | Suicide planning past 12 months | | | Suicide attempt past 12 months | | |
|  | AOR (95% CI) | AOR (95% CI) | AOR (95% CI) | AOR (95% CI) | AOR (95% CI) | AOR (95% CI) |
| "Nutrition deprivation" (2Q vs 1Q) ^a^ | 1.07 (0.98-1.18) | 1.07 (0.97-1.17) | 1.56 (0.82-2.95) | 1.12 (1.00-1.26) | 1.11 (0.98-1.25) | 2.40 (1.03-5.57) |
| "Nutrition deprivation" (3Q vs 1Q) ^a^ | 1.25 (1.12-1.39) | 1.24 (1.12-1.38) | 1.55 (0.75-3.19) | 1.45 (1.28-1.64) | 1.42 (1.25-1.61) | 3.45 (1.47-8.09) |
| "Nutrition deprivation" (4Q vs 1Q) ^a^ | **1.34 (1.22-1.47)** | **1.33 (1.21-1.46)** | **2.09 (1.09-4.02)** | **1.48 (1.31-1.67)** | **1.44 (1.27-1.63)** | **3.85 (1.66-8.94)** |
| "Unhealthy food consumption" (2Q vs 1Q) ^b^ | 1.02 (0.93-1.13) | 1.01 (0.92-1.12) | 1.48 (0.74-2.97) | 0.94 (0.83-1.06) | 0.95 (0.84-1.07) | 0.73 (0.33-1.64) |
| "Unhealthy food consumption" (3Q vs 1Q) ^b^ | 1.12 (1.00-1.24) | 1.12 (1.01-1.25) | 0.91 (0.43-1.94) | 1.14 (1.00-1.29) | 1.15 (1.01-1.31) | 0.66 (0.28-1.55) |
| "Unhealthy food consumption" (4Q vs 1Q) ^b^ | **2.04 (1.86-2.23)** | **2.01 (1.83-2.20)** | **3.26 (1.79-5.95)** | **2.13 (1.91-2.37)** | **2.09 (1.87-2.34)** | **2.95 (1.57-5.55)** |
| "Nutrition deprivation" + "Unhealthy food consumption"  (2Q vs 1Q) | 1.12 (1.01-1.23) | 1.11 (1.00-1.22) | 1.77 (0.80-3.93) | 1.19 (1.05-1.35) | 1.18 (1.04-1.33) | 1.83 (0.76-4.37) |
| "Nutrition deprivation" + "Unhealthy food consumption"  (3Q vs 1Q) | 1.27 (1.15-1.40) | 1.25 (1.14-1.38) | 2.41 (1.17-4.97) | 1.34 (1.18-1.51) | 1.32 (1.17-1.49) | 2.12 (0.96-4.69) |
| "Nutrition deprivation" + "Unhealthy food consumption"  (4Q vs 1Q) | **2.13 (1.94-2.34)** | **2.10 (1.91-2.31)** | **3.78 (1.83-7.81)** | **2.50 (2.23-2.81)** | **2.44 (2.17-2.74)** | **5.13 (2.38-11.10)** |
| Models were adjusted for sex, school grade, socioeconomic status, academic achievement, and family Cohabitation.  a) "Nutrient deprivation" score [range: 4-29] is composite score comprised of breakfast skipping [1-8], and vegitable [1-7], milk [1-7], and fruit deprivation [1-7] as a result of PCA analysis.  b) "Unhealthy food consumption" score [range: 6-36] is composite score comprised of carbonated [1-7] and uncarbonated sugar-based beverage [1-7], and consumption of instant noodle, caffeinated drink, snacks and fast food [1-7].  AOR, adjusted odds ratio; CI, confidence interval; Q, quartile.  Statistical significant tested where P < 0.01. | | | | | | |
